# Characterization and comparison of the bacterial communities of rhizosphere and bulk soils from cadmium-polluted wheat fields

**DOI:** 10.7717/peerj.10302

**Published:** 2020-11-04

**Authors:** Li Song, Zhenzhi Pan, Yi Dai, Lin Chen, Li Zhang, Qilin Liao, Xiezhi Yu, Hongyan Guo, Guisheng Zhou

**Affiliations:** 1 Joint International Research Laboratory of Agriculture and Agri-Product Safety, Jiangsu Key Laboratory of Crop Genomics and Molecular Breeding, Co-Innovation Center for Modern Production Technology of Grain Crops, Yangzhou University, Yangzhou, Jiangsu, China; 2 College of Bioscience and Biotechnology, Yangzhou University, Yangzhou, Jiangsu, China; 3 State Key Laboratory of Pollution Control and Resource Reuse, School of Environment, Nanjing Univerity, Nanjing, Jiangsu, China; 4 Geological Survey of Jiangsu Province, Nanjing, Jiangsu, China

**Keywords:** Wheat, 16S rRNA amplicon sequencing, Cadmium, Microbial community, Rhizosphere

## Abstract

Cadmium pollution is becoming a serious problem due to its nondegradability and substantial negative influence on the normal growth of crops, thereby harming human health through the food chain. Rhizospheric bacteria play important roles in crop tolerance. However, there is little experimental evidence which demonstrates how various cadmium concentrations affect the bacterial community in wheat fields including rhizosphere microorganisms and nonrhizosphere (bulk) microorganisms. In this study, 16S rRNA amplicon sequencing technology was used to investigate bacterial communities in rhizosphere and bulk soils under different levels of pollution in terms of cadmium concentration. Both the richness and diversity of the rhizosphere microorganism community were higher under nonpolluted soil and very mild and mild cadmium-contaminated soils than compared with bulk soil, with a shift in community profile observed under severe cadmium pollution. Moreover, cadmium at various concentrations had greater influence on bacterial composition than for the nonpolluted site. In addition, redundancy analysis (RDA) and Spearman’s analysis elucidated the impact of exchangeable Cd and total Cd on bacterial community abundance and composition. This study suggests that cadmium imposes a distinct effect on bacterial community, both in bulk and rhizosphere soils of wheat fields. This study increases our understanding of how bacterial communities in wheat fields shaped under different concentrations of cadmium.

## Introduction

It is well known that heavy metals accumulate excessively in agricultural soils due to increasing heavy-metal loading from industrial contamination, sewage-water irrigation, and the application of sewage-sludge and heavy-metal-containing fertilizers (Dong, Cui & Liu, 2001; Qayyum et al., 2017). Heavy metals not only cause the deficiency and imbalance of nutrients in field-crop plants grown in polluted soils but also are transferred to the food chain through plants, which endangers food security and ecological safety (Adrees et al., 2015; Sharma & Archana, 2016). Cadmium (Cd) was reported to be a highly toxic heavy metal among nonessential metals that impairs plant growth and microorganism diversity in soils, even at low concentrations (Bertin & Averbeck, 2006; Rizwan et al., 2016; Rizwan et al., 2017).

Recently, the effects of Cd on microbial communities, such as the decrease of microbial taxonomic species and the change of microbial compositions in soil have received research attention (Vig et al., 2003; Krujatz et al., 2012; Feng et al., 2018b). Luo et al. (2018) reported that high Cd level of agricultural paddy soil displayed lower bacterial community diversity than low Cd level sites. Lu, Xu & Chen (2013) found significant negative impacts of Cd on soil microbial activity and community structure in a greenhouse experiment. As the microbial community not only adapts to the soil environment but also affects the accumulation of metals in plants, characteristics of microbial community structure in the contaminated environment are often used as indicators of soil quality (Schloter, Dilly & Munch, 2003; Sessitsch et al., 2013). Several studies have previously shown that bacteria can mediate the alleviation of heavy-metal stress, and the decreased absorption and accumulation of metals in plant tissue through metal exclusion, extrusion, accommodation, biotransformation, methylation, and demethylation (Hardoim, Overbeek & Elsas, 2008; Etesami, 2018). Plant-growth-promoting rhizobacteria (PGPRs) have been recognized as a crucial coevolutionary factor that developed resistance or tolerance towards heavy metals through intrinsic or induced mechanisms and have been used by plants to ameliorate heavy-metal stress (Madhaiyan, Poonguzhali & Sa, 2007; Rajkumar et al., 2012; Singh et al., 2019). For example, the inoculation of *Pseudomonas aeruginosa* and *Burkholderia gladioli* enhanced Cd tolerance in the seedlings of *Lycopersicon esculentum* (Khanna et al., 2019). Moreover, many reports have shown that bacterial-community structure and diversity respond to direct heavy-metal contamination in soil (Abdu, Abdullahi & Abdulkadir, 2017; Luo et al., 2018; Hou et al., 2018). For example, the bacterial community of soils contaminated with Pb, Zn, and Cr significantly differ, with Zn decreasing both diversity and species richness at the species and family levels, while plant-species richness was not correlated with bacterial diversity (Golebiewski et al., 2014). Bacteria corresponding to two phyla (Proteobacteria and Firmicutes) were identified as being highly resistant to heavy-metal content in mines (Zhao et al., 2019). Thus, the critical community composition and activities of a microbial population could serve as indicators for improving soil quality and ecosystem restoration (Yin et al., 2000; Banning et al., 2011; Honeker et al., 2019).

Previous studies have shown that heavy-metal uptake by crops is closely related to soil type, soil-contamination level, and the availability of heavy metal in soils (Williams et al., 2009; Harris & Taylor, 2013; Feng et al., 2018a). Higher capacity for Cd accumulation and translocation was found in wheat, which may enhance Cd accumulation in wheat grains (Tavarez, Macri & Sankaran, 2015). Cd-induced toxicity in wheat leads to reduced seed germination and root and shoot growth, changes nutrient ratios in tissue, and causes plant death, especially for higher levels of Cd in the growth medium (Ci et al., 2010; Ahmad et al., 2013; Yourtchi & Bayat, 2013; Rizwan et al., 2016). Therefore, alleviating Cd toxicity and minimizing its uptake in wheat has become a serious problem that needs to be urgently solved. Research on plant–bacteria interactions revealed that plants can shape their rhizosphere bacterial communities and recruit environmental-stress-resistant bacteria (Berendsen, Pieterse & Bakker, 2012). Dissection of the bacterial community in the rhizosphere of *Baphicacanthus cusia* (Nees) Bremek showed that it was remarkably different from that in the bulk soil, and *B. cusia* can specifically recruit microbes from the bulk soil and host them in the rhizosphere (Zeng et al., 2018). The rhizosphere of rice-root-associated microbiomes was found to be distinct from the endosphere (root interior) and rhizoplane (root surface) (Edwards et al., 2015).

The investigation and application of soil microorganisms with proven ability to remediate and tolerate heavy toxicity will reduce soil contamination (Jing et al., 2014; Ojuederie & Babalola, 2017). It was reported that the shaping of rhizosphere correlated with wheat cultivars (Mahoney, Yin & Hulbert, 2017). However, there has been little consideration and knowledge on how wheat-rhizobacterium community features are shaped under various Cd concentrations. Therefore, a better understanding of the diversity of rhizobacteria comparing with those in nonrhizosphere soil (bulk soil) may be an important strategy to eliminate or reduce the effects caused by Cd in various environmental compartments. Recently, high-throughput sequencing technology has provided a powerful tool to explore those complex microbial communities and their shifting profiles under Cd contamination. In this study, the microbial responses to various concentrations of heavy-metal contamination were explored in terms of the composition, diversity, structure, and functional potential of bacterial communities in rhizosphere and nonrhizosphere soils through the sequencing of 16S rRNA gene amplicons.

## Materials & Methods

### Field description and soil collection

The sampling area for this study was about 50 km^2^ (Location coordinates: longitude 119.633, latitude 31.399) in Xushe Township of Yingxing, Jiangsu province, China. The annual average temperature and average altitude of Yixing are 16.7 °C and 5–8 m, respectively. Yixing has a subtropical monsoon climate. The Cd content has been monitored for over 10 years in this area. A total of 74 sampling sites were set up to monitor soil physical and chemical properties (Fig. S1). Rice and wheat are alternately planted in each sampling site every year. Prior to the start of the project, permission was obtained from the landowner (Zuliang Jiang, Jiang Farms, Yifeng Village, YiXing) to access the site and monitor soil quality. Four field sites were selected according to cadmium-contamination levels, including a nonpolluted site (CK) and three sites with very mild contamination (VMC), mild contamination (MC), and severe contamination (SC), respectively. The size of each sampling plot was about 300 m^2^. Six replicate samples were systematically collected across each plot and tested. Sample collection was performed under well-drained soil conditions. Within each field environment, rhizosphere and bulk soils were collected as described by Edwards et al. (2015). Therefore, a total of 48 samples (4 fields X 6 replicate X 2 soil compartment of bulk or rhizosphere) were collected. Briefly, soils were collected from the top 10 cm of the profile, and plant tissue was removed through a two mm sieve for the collection of bulk soil. Loose soil on the surface of wheat (*Triticum aestivum* L.) root was gently removed, and firmly attached soil was collected as rhizosphere soil. Each biological replicate was mixed from more than 30 plants. Soil samples were transported to the lab on dry ice and then stored at −80 °C until being used for molecular analyses. Another fraction of the bulk soil was air-dried for soil geochemical analysis.

### Soil chemical characterization and Cd contaminant fraction analysis

After the soil samples were air-dried, animal and plant residues were removed. Then, soils were crushed with a rubber hammer and screened using a two mm sieve. The screened soil sample was thoroughly mixed and placed into a plastic sample bag for geochemical analysis. Hydrometer method was used to determine the particle size distribution of grained soils passing two cm sieve. Ten grams of dry soil was placed in a 100 mL beaker containing 25 mL of water. The mixture was stirred for 5 min and then allowed to stand for 1 h to equilibrate. The soil pH value was then measured using a calibrated pH meter (Mettler-Toledo FiveEasy Plus, Columbus, USA) on the basis of an agricultural professional standard (NY/T 1377-2007). Cation-exchange capacity (CEC) was determined using NaOAc flame photometry following the agricultural professional standard NY/T 295-1995. Soil organic matter (SOM) was determined by the K_2_Cr_2_O_7_ colorimetric method (agricultural professional standard NY/T1121.6-2006). Total phosphorus (TP) and available phosphorus (AP) were extracted with 0.5 mol/L NaHCO_3_ and measured using a spectrophotometer and the agricultural/forestry professional standards (NY/T 88-1988 and LY/T1232-2015). Total nitrogen (TN) and hydrolyzable nitrogen (HN) were estimated according to agricultural/forestry professional standards (NY/T53-1987 and LY/T1228-2015). Total Cd was determined by the graphite-furnace atomic-absorption spectrophotometry following China’s national standard (GB/T17141-1997). Exchangeable Cd in soils was extracted using the Tessier procedure (Tessier, Campbell & Blsson, 1979).

### DNA extraction and amplification

Genomic DNA was extracted from 3 g of well-mixed soil for each sample using the DNA Extraction Kit (DNeasy PowerSoil Kit, Qiagen, Cat#: 12888-100) and the manufacturer’s instructions were followed. The DNA concentration was further measured with NanoDrop and the quality checked using agarose gel. Then, the sample was diluted to 1 ng/µL and stored at −20 °C until further processing. The diluted DNA was used as a template for PCR amplification of bacterial 16S rRNA genes with barcoded primers and Takara Ex Taq (Takara, Cat#: PR001Q). For bacterial-diversity analysis, the V3–V4 variable regions of 16S rRNA genes were amplified with universal primers 343F (5′-TACGGRAGGCAGCAG-3′) and 798R (5′-AGGGTATCTAATCCT-3′).

### Library preparation and sequencing

Amplicon quality was visualized using gel electrophoresis, purified with AMPure XP beads (Agencourt), and amplified for another round of PCR. After purification with the AMPure XP beads again, the final amplicon was quantified using the Qubit dsDNA assay kit (Life Technologies, Cat#: Q32852). The same amounts of purified amplicon from each sample were pooled together for subsequent sequencing. High-throughput sequencing of amplicons was performed on the Illumina MiSeq platform at OEbiotech Company (Shanghai, China).

### Data analysis and access

Paired-end reads were then preprocessed using Trimmomatic software to detect and remove ambiguous bases (N) (Bolger, Lohse & Usadel, 2014). A sliding-window trimming approach was used to excise low-quality sequences. The FLASH software was used to assemble reads according to the following parameters: 10 bp of minimal overlapping, 200 bp of maximal overlap, and 20% of maximal mismatch rate (Reyon et al., 2012). High-quality reads were then obtained using QIIME (1.8.0) and Vsearch software with 97% similarity cut-off threshold (Caporaso et al., 2010; Edgar et al., 2011; Rognes et al., 2016). All representative reads were annotated and blasted against Silva database version 123 (or Greengens) (16S rDNA) using an RDP classifier (confidence threshold was 70%) (Altschul et al., 1990; Wang et al., 2007). The raw sequence reads of our 16S rRNA gene were deposited at National Center for Biotechnology Information (NCBI) Short Read Archive (SRA, http://www.ncbi.nlm.nih.gov/sra/) under the accession number PRJNA661129.

### Statistical analysis

PD whole tree as well as Chao1 and Shannon indices were applied to evaluate alpha diversity. Error bars were SE and *P* values were from two-tailed Student’s *t*-test. Bray–Curtis distance was used to evaluate beta diversity in different soil samples. All analyses, from chimera checking to alpha- and beta-diversity calculation, were performed using QIIME (1.8.0) (Caporaso et al., 2010). Principal-component analysis (PCA) was conducted using the ade4 and ggplot2 packages of R software. Statistical analysis of the relative abundance of the genera as well as diversity indices and estimators were performed using R (v3.2.2). Linear-discriminant-analysis effect size (LEfSe) was performed to determine differential features between groups (Segata et al., 2011). The threshold on logarithmic score (LDA) for discriminative features was set to 4.0. Redundancy analysis (RDA) was conducted to measure the influence of contamination fractions on microbial-community structure by using OmicShare tools, a free online platform for data analysis (https://www.omicshare.com/tools). For Spearman’s correlation analysis, the Spearman correlation coefficient of the species and environmental factors were first calculated using the corr.test function of the psych packet in R, and significance was tested and visualized using the pheatmap function in the pheatmap package.

## Results

### Cd geochemical parameters and concentration in sampling sites

The parameter pH was first analyzed for all bulk soil samples, and results suggest that the soil was weakly acidic or close to neutral (pH 5.44–6.79). Analysis of soil texture indicated that most of the wheat field was acidic silty clay loam (30 ± 9% clay, 54 ± 8% silt, 16 ± 4% sand). The range of soil cation-exchange capacity (CEC) was 15.87–20.86 cmol kg^−1^. Organic-matter content (SOM) in the soil varied in the range of 18.5–42.6 g kg^−1^. Soil total nitrogen (TN, 0.105–0.2 g/kg), hydrolytic nitrogen (HN, 122–349 g/kg), total phosphorus (TP, 0.047–0.087 g/kg), and available phosphorus (AP, 9.83–50.3 g/kg) were assessed (Table S1). Therefore, the soil fertility grade in these sampling sites was high.

The Cd concentration greatly differed for each collected sample site, in which the content of total Cd ranged from 0.17 to 14.64 mg kg ^−1^, while the exchangeable Cd was within the range of 0.07–7.28 mg kg ^−1^ (Table S2). Soil samples from the contaminated areas showed significantly elevated concentrations of both total and exchangeable Cd. Exchangeable Cd content was about one-half to two-thirds less than total Cd content and significant difference was found in MC and SC sites. Moreover, either total or exchangeable Cd in rhizosphere soil was lower than that in bulk soil, but only significant difference was found under severe pollution condition. According to the risk-control standard of agricultural-land pollution for soil environmental quality (China national standard GB15618-2018) in China, values for the VMC and MC sites were higher than the risk-screening value, but lower than the risk-intervention value for the soil contamination of agricultural land. However, the value of the SC site was much higher than the risk-intervention value.

### Global sequencing data and microbial diversity across all sampling sites

The high-throughput sequencing of 16S rRNA amplicons yielded 13,622–41,511 clean and valid tags (average length 427.83–434.22 bps) in all 48 samples after filtering out low-quality reads and trimming by removing sequences corresponding to adapters, barcodes, primers, and chimeras. The rarefaction curves for the Shannon index reached an asymptote (Fig. S2), indicating that the current numbers of sequence reads were sufficient to capture bacterial diversity in those soils. Good’s coverage estimator ranged from 0.91 to 0.93, indicating that the sequencing captured the dominant phylotypes (Table S3).

**Figure 1:**
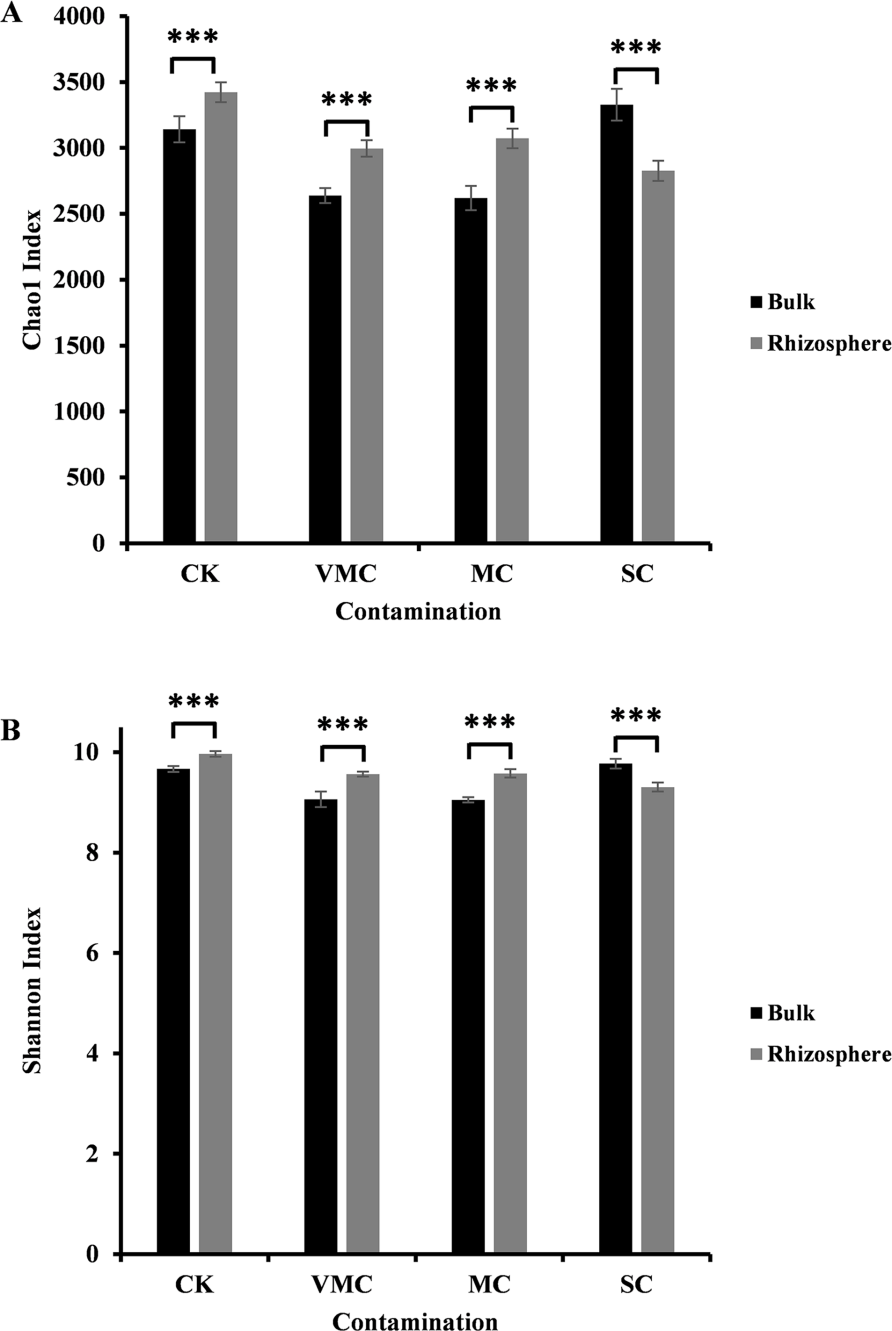
Alpha-diversity comparisons of microbiota richness and diversity as measured by (A) Chao1 and (B) Shannon indices observed in the microbiota of bulk (black) and rhizosphere (grey) soils (six samples per each site). Each value is an average of each site coming from various contamination contents (CK, no contamination; VMC, very mild contamination; MC, mild contamination; SC, severe contamination). *P* < 0.001, ∗∗∗.

In our study, the Chao1 and Shannon indices indicated a significant difference between rhizosphere and bulk soils. As indicated in Fig. 1, either community richness (Chao1 index, Fig. 1A) or abundance (Shannon index, Fig. 1B) of rhizosphere microorganisms under CK, VMC, and MC pollution levels was higher than that of bulk microorganisms. Interestingly, rhizosphere soils under severe Cd pollution conditions contained less bacterial-community richness and abundance than those of bulk soils. With an increased pollution degree, the Chao1 and Shannon indices of rhizosphere microorganisms decreased, but the Chao1 and Shannon indices of bulk-soil microorganisms under severe pollution were higher than those of other soil conditions. These results indicate that severe Cd pollution results in significant differences in the bacterial-community structure between bulk and rhizosphere soils.

**Figure 2:**
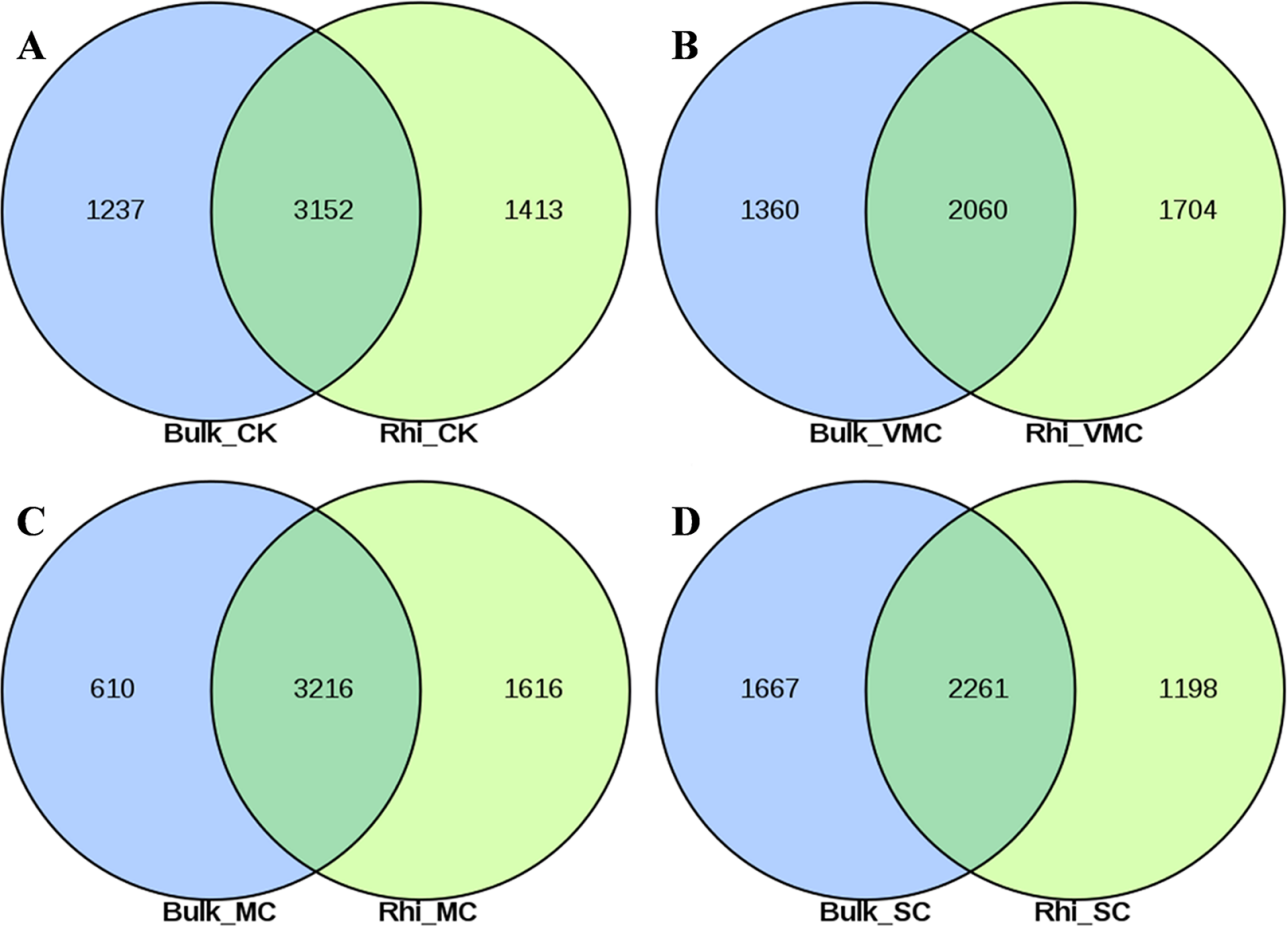
Venn diagrams of shared operational taxonomic units (OTUs) between bulk and rhizosphere soils under different contamination levels. (A) Without cadmium-contamination (B) Very mild cadmium-contamination (C) Mild cadmium-contamination (D) Severe cadmium-contamination condition. OTUs counted only when they were biologically replicated at least once.

A total of 7,470 and 7,541 operational taxonomic units (OTUs) were obtained on the basis of high-quality sequencing reads (97% sequence similarity) in bulk- and rhizosphere-soil samples, respectively (Figs. S3A, S3B). The Venn diagram shows that these OTUs shared 48.2%–68.1% similarity between bulk and rhizosphere soils under the same pollution levels, while 615–1,398 OTUs were only enriched in the bulk soils and 778–1,923 OTUs were unique to the rhizosphere soils (Figs. 2A–2D). Overlaps between different contamination levels were determined for bulk and rhizosphere soil and further compared. As indicated in Fig. S3, the Venn diagram showed that 2,238 core OTUs existed in the bulk soil, and 2654 core OTUs existed in rhizosphere soil (Figs. S3A, S3B). The trend of observed OTU numbers in the bulk group was SC (2130) >  CK (2004) >  VMC (1695) >  MC (1625); in rhizosphere soils, this trend was CK (2153) >  MC (1923) >  VMC (1863) >  SC (1835) (Table S3). The unique OTUs in bulk soils were further observed, with 590(7.9%), 161(2.2%), 631(8.4%), and 577(7.7%) unique OTUs in CK, VMC, MC, and SC groups, respectively. Unique OTUs in rhizosphere soils were also observed, with 540(7.2%), 298(4%), 438(5.8%), and 461(6.1%) unique OTUs in the CK, VMC, MC, and SC groups, respectively.

### Bacterial diversity and distribution in community structure between bulk and rhizosphere soils

**Figure 3:**
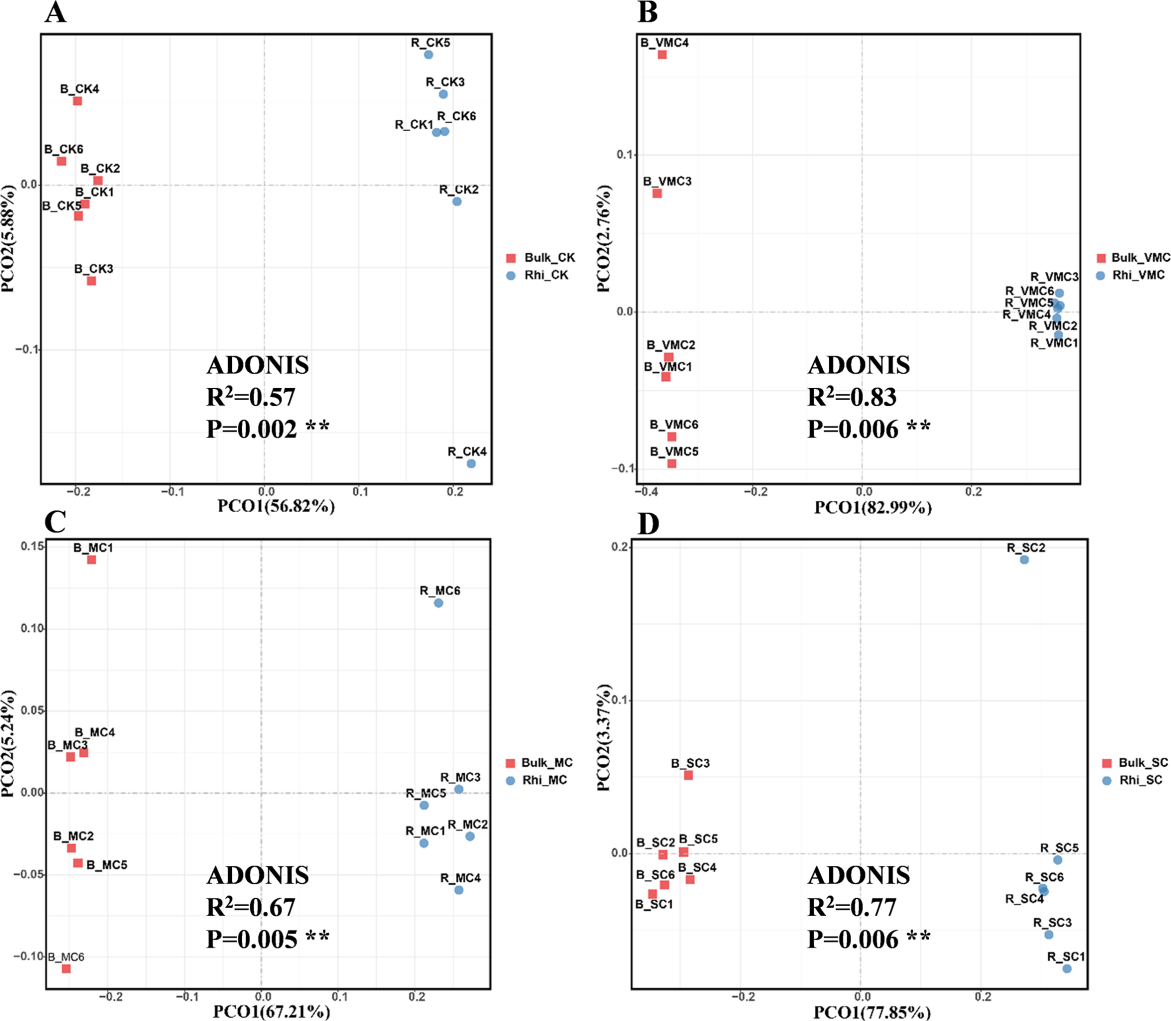
Bray–Curtis distance principal co-ordinate analysis (PCoA) of bacterial communities with ADONIS statistics *P*-values (*P* < 0.01) between bulk and rhizosphere soils under various pollution levels. Two different soil samples were separately colored, with the same coloring scheme applied to all four graphs.

Beta diversity is an indicator of diversity in microbial communities among groups. Principal co-ordinate analysis (PCoA) was performed to investigate separation patterns of microbial communities between bulk and rhizosphere soils. First, using the Bray–Curtis similarity index, significant differences in beta diversity were observed between bulk and rhizosphere soil for the four groups (*P* = 0.002, 0.06, 0.005, and 0.006, respectively; Fig. 3). Second, ADONIS statistics showed an R^2^ value of 0.57 between bulk and rhizosphere soils at the CK site. However, a higher R^2^ value of 0.67–0.83 was found between bulk and rhizosphere soils at the three other polluted sites (Fig. 3). These results not only suggest that microbial communities were distinct between bulk and rhizosphere soils, but also indicate that different degrees of Cd pollution have an enhanced effect on altering the diversity of microbial composition compared to the CK site. In addition, PCoA-plot shows the difference among bulk soils or rhizosphere soils under various Cd concentrations. PCoA resulted in a 3-dimensional solution in bulk soils, PC1, PC2 and PC3 accounted for 50.81, 23.72 and 8.94%, respectively (Fig. S4A). In rhizosphere soils, PC1, PC2 and PC3 accounted for 48.64, 20.17 and 13.02%, respectively (Fig. S4B). Overall, these results indicate a clear division among bulk soils or rhizosphere soils.

**Figure 4:**
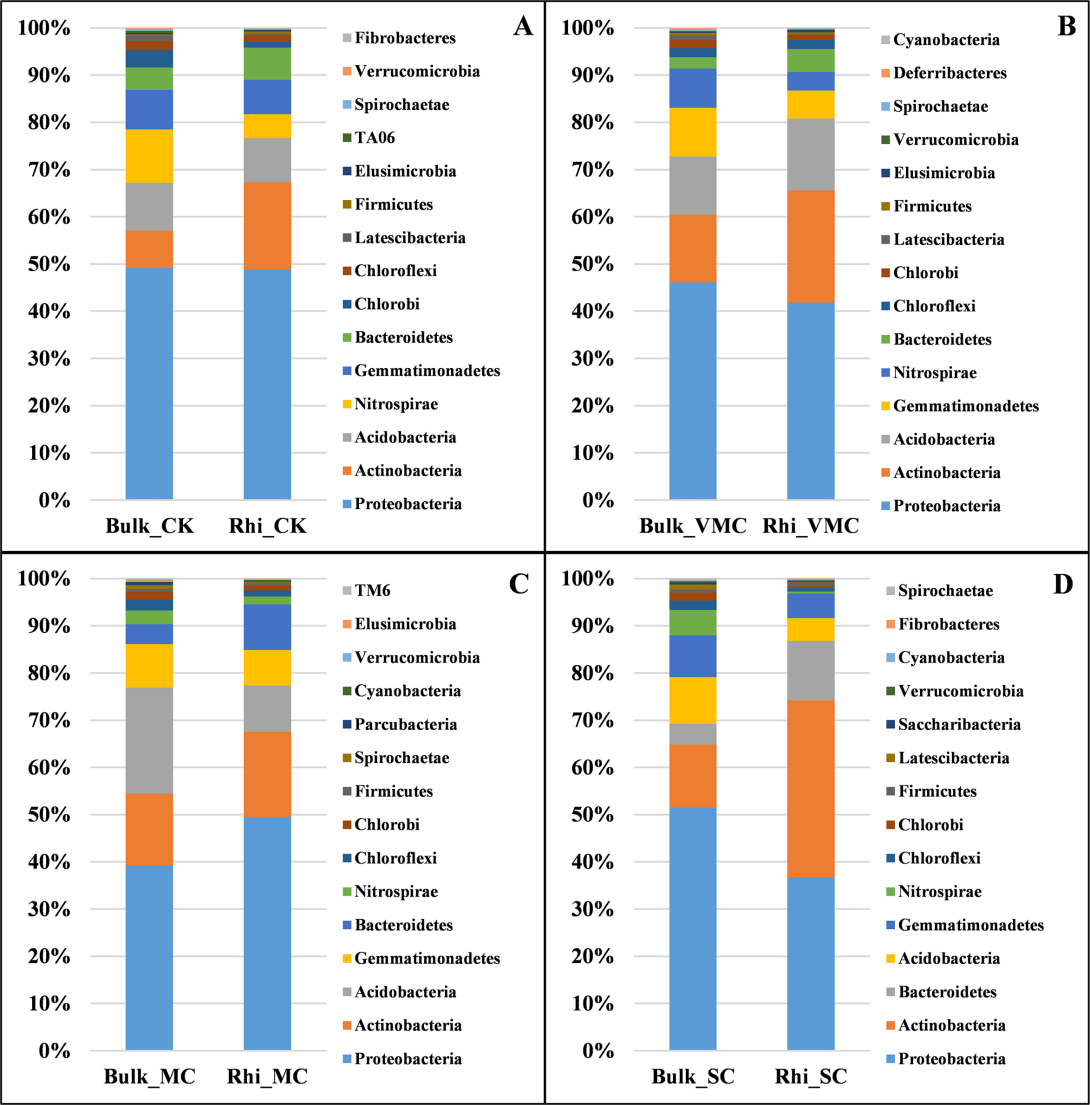
Bacterial-community composition between bulk and rhizosphere soils based on relative abundance of the top 15 bacterial phyla under various contamination conditions. (A) Without cadmium-contamination (B) Very mild cadmium-contamination (C) Mild cadmium-contamination (D) Severe cadmium-contamination condition.

To further analyze bacterial structure and composition, the relative bacterial abundance in bulk and rhizosphere soils at different taxonomic levels was examined. The top 15 bacteria in terms of relative abundance are presented in this study. At the phylum taxonomic level, Proteobacteria had the highest abundance in both bulk and rhizosphere soils under various pollution levels. Species of Actinobacteria were the second most abundant bacteria in both bulk and rhizosphere soils, showing an increasing proportion in rhizosphere soil, especially in rhizosphere soil under SC conditions. The microbiome in bulk and rhizosphere soils was characterized by a prominent Acidobacteria, Nitrospirae, Gemmatimonadetes, and Bacteroidetes (Fig. 4).

There were marked differences in the proportions of various classes across soils. Deltaproteobacteria corresponded to the largest class in bulk soil under CK, VMC, and SC conditions, and Acidobacteria were the most abundant in bulk soil under MC conditions. Bulk soil at the MC site had a significantly greater proportion of Acidobacteria, whereas it was mostly depleted in rhizosphere soil compared with that of bulk soil. The reduction in the relative abundance of these phyla and classes under SC conditions across all contamination levels is consistent with the observation that microbial diversity decreases with an increase in pollution level (Fig. 4 and Fig. S4).

### Correlation between Cd concentration levels and microbial communities

**Figure 5:**
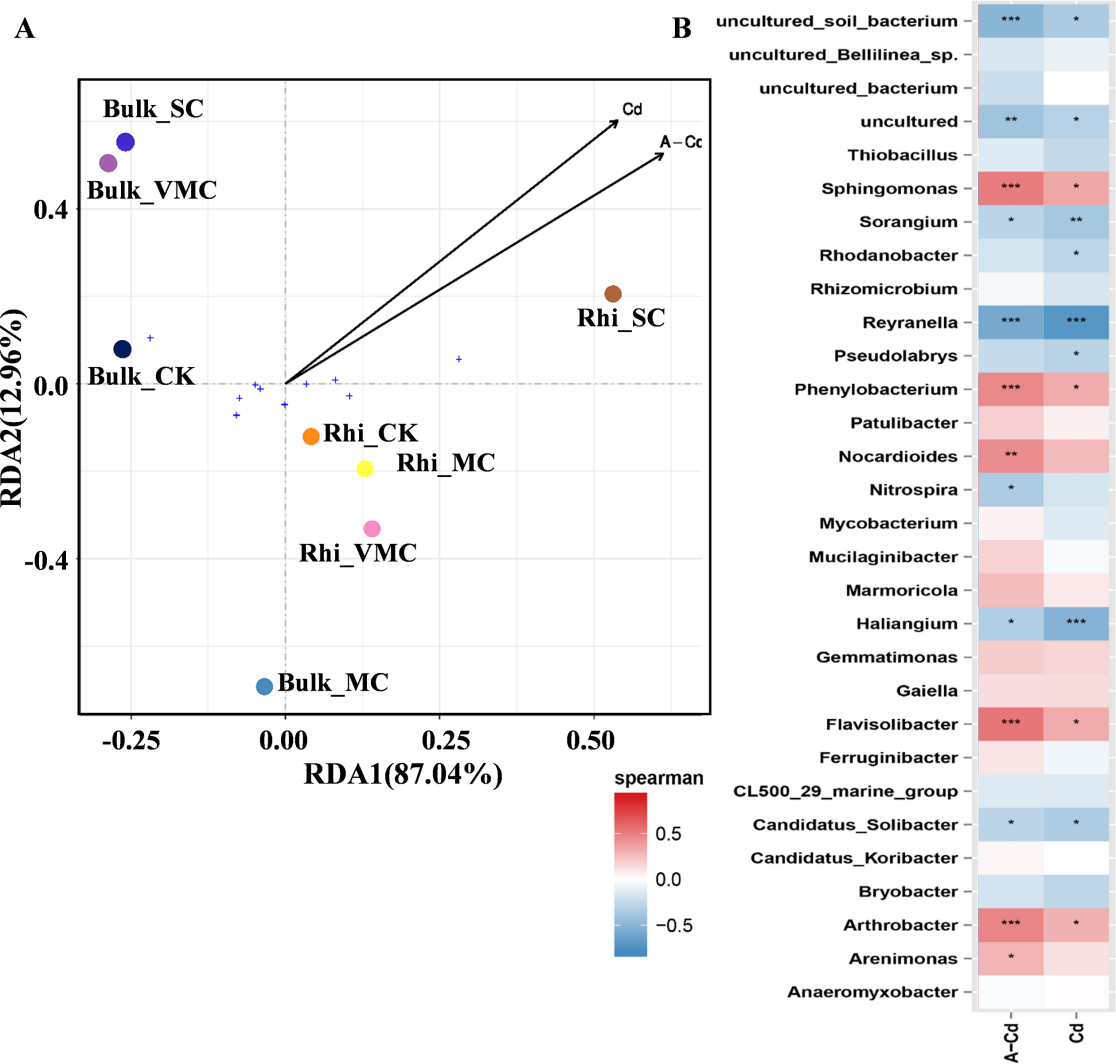
Redundancy analysis (RDA) and correlation of bacterial community structures depicting relationships between samples or species distribution and cadmium content. (A) Arrows, direction, and magnitude of heavy-metal parameters associated with bacterial-community structures at class taxa. The top 10 most abundant classes across all samples were selected to represent the overall microbial community. The *x*- and *y*-axes explain 87.04% and 12.96% of the variation, respectively. The length of arrows in the RDA plot correspond to the strength of the correlation between Cd and community structure. (B) The Spearman’s correlation between cadmium content and bacterial-community structures based on the genus as assessed using Spearman’s correlation analysis. Cd, total Cd; A-Cd, exchangeable Cd. ^∗^*P* < 0.05, ^∗∗^*P* < 0.01, ^∗∗∗^*P* < 0.001. Red represents a positive correlation, blue represents a negative correlation.

Redundancy analysis (RDA) was used to quantify the relative influence of Cd concentration, including exchangeable and total Cd, on microbial-community abundance and composition (Fig. 5A). The results show that for the first and second axes, the weighted values explain 87.04% and 12.96% of the difference, respectively. Bulk samples were clustered together but well separated from rhizosphere samples. Especially, bacterial community composition in rhizosphere soil varied significantly under high Cd concentration compared with other rhizosphere soils as reflected by the axis 1. As reflected by the axis 2, there was a big distinction between Bulk_MC and other bulk samples, while an approaching trend between Bulk_VMC and Bulk_SC was found. The results suggested that the effect of higher concentration of total Cd and exchangeable Cd on the bacterial communities in rhizosphere soil was greater than that of bulk soil.

The heatmap based on Spearman’s analysis was used to analyze the relationship between Cd concentration and microbial abundance at the genus level (top 30). *Arthrobacter*, *Flavisolibacter*, *Phenylobacterium*, and *Sphingomonas* were significantly positively correlated with exchangeable Cd. However, *Reyranella* was significantly negatively correlated with the concentrations of exchangeable and total Cd (Fig. 5B).

### Functional-capacity profiling prediction of bulk and rhizosphere microbiomes under severe pollution conditions

**Figure 6:**
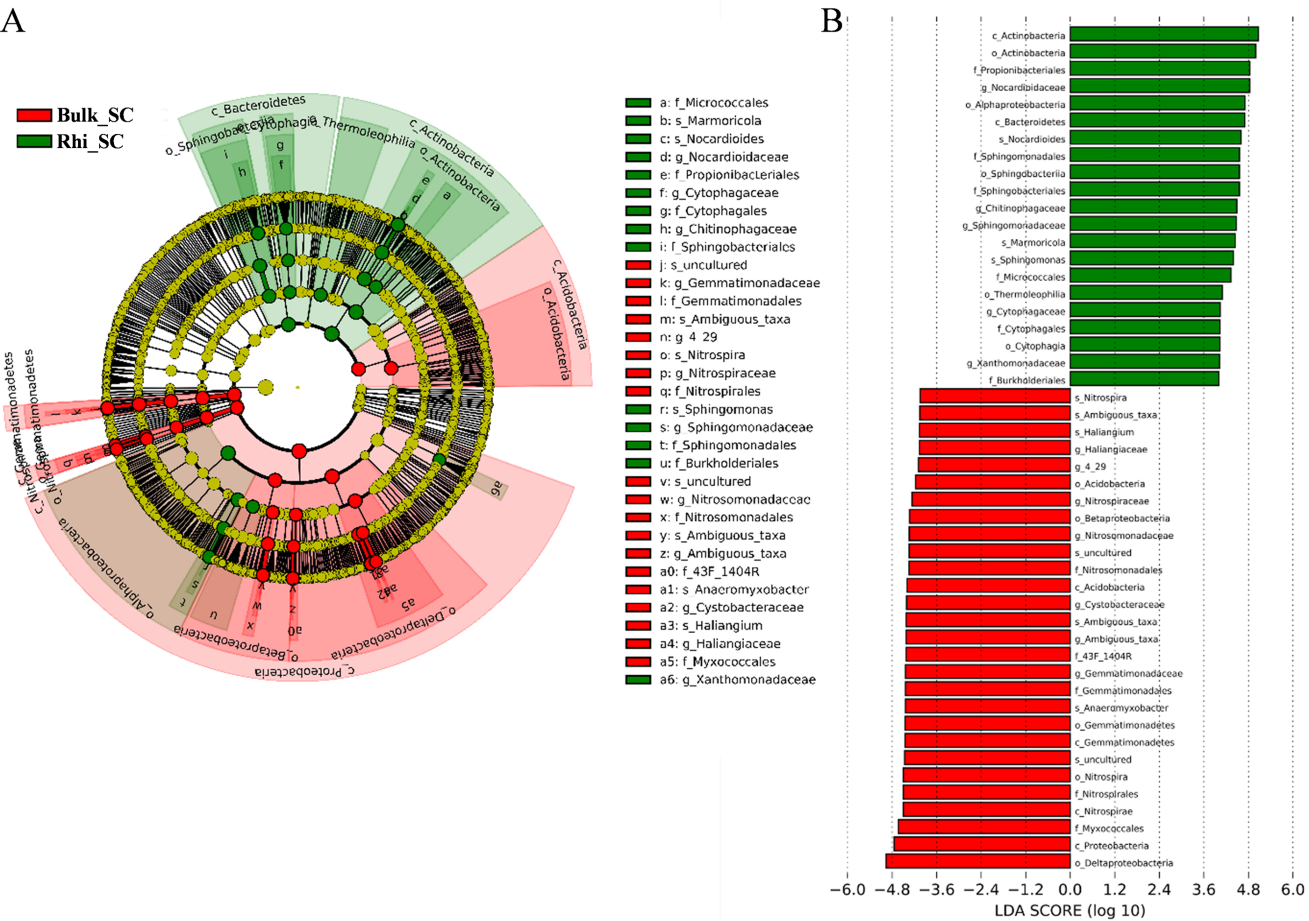
Abundance histogram of major taxonomic units that were significant in bulk and rhizosphere soils under severe cadmium contaminations condition using linear-discriminant-effect size analysis (LEfSe). (A) Cladogram of bacterial communities between bulk and rhizosphere soils under severe cadmium contamination. Small circles and colored areas represent the abundance of taxa in each respective group (red, bulk soil; green, rhizosphere soil). Yellow circles, indicate nonsignificant differences in abundance between both soil samples for a particular taxonomic group. (B) LDA scores of biomarker bacteria, shown as horizontal bars for biomarker bacteria with an LDA score (log10) > 4 and with alpha value of 0.01.

Linear-discriminant-effect size analysis (LEfSe) was used to evaluate differentially represented OTUs between bulk and rhizosphere soils under severe pollution conditions (Fig. 6A). Only taxa meeting an LDA significance threshold of 4.0 ((log10) >  4, *P* < 0.01) are shown in the histogram (Fig. 6B). Analysis revealed that at the severe pollution site, proportions of species corresponding to the genera *Nitrospira*, *Ambiguous* _*taxa*, *Sphingomonas*, *Anaeromyxobacter*, and *Haliangium* were elevated in bulk soil, whereas most highly represented bacterial species in rhizosphere soil under severe pollution were *Marmoricola*, *Nocardioides,* and *Sphingomonas*. Genera of *Chitinophagaceae* were found to predominate in rhizosphere soil, whereas genera belonging to *Cystobacteraceae* were found to predominate in bulk soil. In addition, the differentially represented OTUs between bulk or rhizosphere soils under different pollution conditions were evaluated. As shown in Figs. S6 and S7, more taxes were found under server pollution conditions in rhizosphere, but less taxes were found under server pollution conditions compared with other conditions. These results may indicate that the effect of server Cd on rhizosphere was greater.

**Figure 7:**
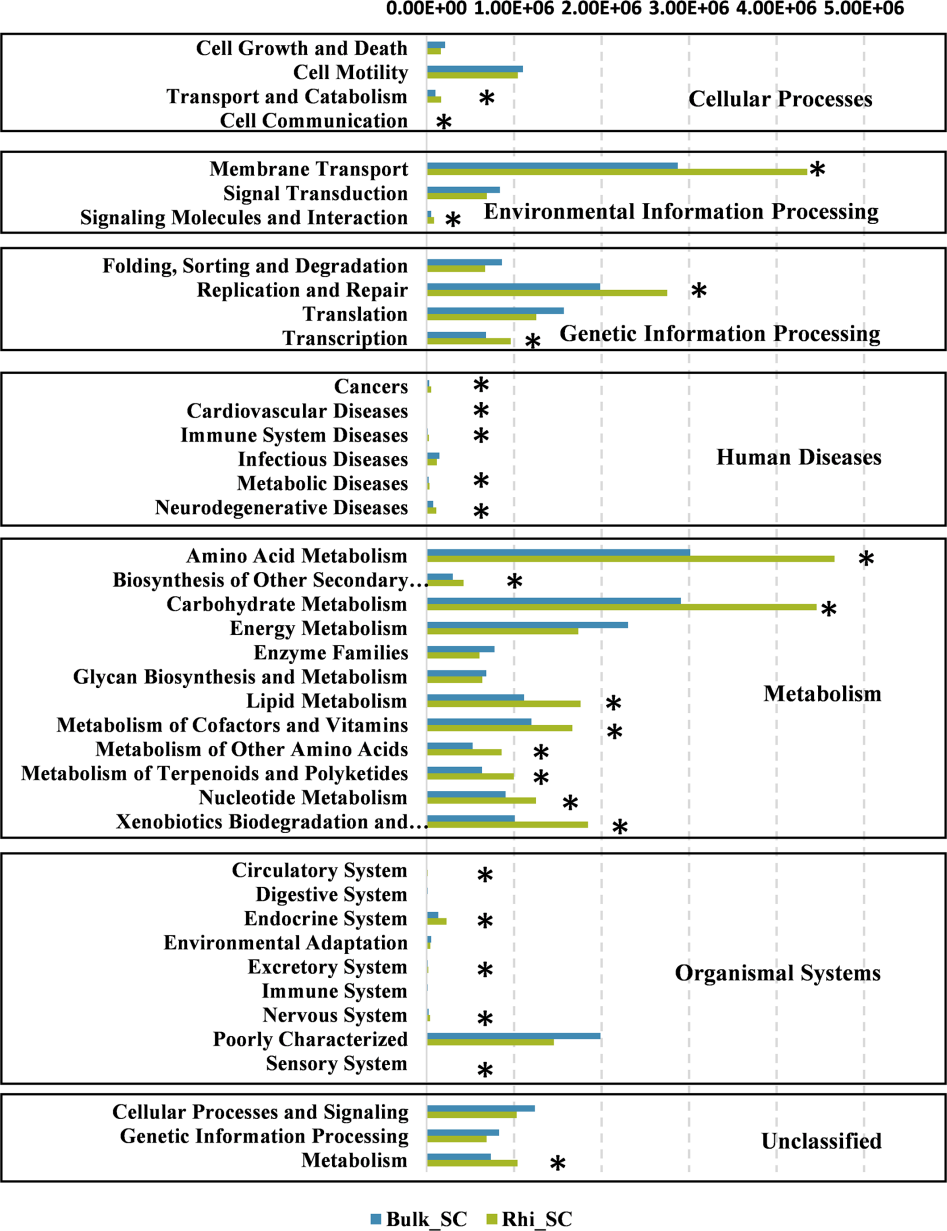
Predicted functions of bacterial communities found in bulk and rhizosphere soils under severe pollution obtained by PICRUSt results at KEGG Level 2. Asterisks (^∗^*P* < 0.05) indicate significant differences between bulk and rhizosphere soils as determined using Kruskal–Wallis testing.

To further determine the predicted metabolic functions of microbial communities between bulk and rhizosphere soils under severe pollution conditions, KEGG metagenome functional prediction of identified OTUs based on 16S rRNA gene sequences was performed using the PICRUSt tool. A total of 41 KEGG pathways possibly related to severe Cd pollution were detected. These results suggest that more OTUs were assigned to processing of environmental- and genetic information in addition to metabolic pathways (Fig. 7). More specifically, 26 KEGG pathways exhibited distinct changes between bulk and rhizosphere soils under severe pollution conditions (*P* < 0.05). Compared with bulk soil, there were significant increases in relative abundances of communities in rhizosphere soil for the above functional categories, with the exception of the sensory-system pathway. In particular, the pathway of carbohydrate and amino acid metabolism was almost 1.5 times more represented in rhizosphere soil than that in bulk soil.

## Discussion

A better understanding of the impact of heavy metals on soil microorganisms not only benefits environmental-pollution control, but also increases crop production. In the present study, we assessed the bacterial communities of bulk and rhizosphere soils in wheat fields with low to high levels of Cd pollution. Distinctive bacterial communities were found for bulk and rhizosphere soils, and these microbes responded differently to Cd pollution at different levels, especially under the severe pollution condition.

### Key role of Cd in determining bacterial-community structure between bulk and rhizosphere soils in wheat fields

Cd contamination has been demonstrated to decrease the number of different taxonomic microbial species in soil and change the soil microbial composition (Feng et al., 2018b; Wang et al., 2019a). Wang et al., (2019a) reported that 10 mg/L Cd (II) reduced microbial diversity and changed the overall microbial-community structure . The research of Shi et al. indicated that the diversity and richness of soil bacteria were significantly increased after continuous amendments (such as lime, and lime mixed with organic manure or phosphate fertilizer) applied in Cd-contaminated soil (Shi et al., 2019). Both the quantity and quality of rhizosphere microorganisms are generally higher than those of nonrhizosphere soil (Wu et al., 2014). However, few available reports detail how different Cd concentrations influence microbial communities. Our results indicate that both the diversity and richness of rhizosphere microorganisms decreased with the increase in cadmium-pollution levels. These results are consistent with previous reports. Moreover, both bacterial diversity and richness in the bulk soil at the SC site were higher than those of CK, VMC, and MC (Fig. 1). Compared with the bulk soil, the high concentration of Cd has a serious effect on the bacterial structure of rhizosphere soil. It was reported that bulk soil, unlike the more active rhizosphere environment, has relatively low rates of nutrient transformation and microbial activity (Ai et al., 2012). Therefore, bacterial communities in bulk and rhizosphere soils receive different feedback under different concentrations of Cd.

The microbial taxa *Nitrospira* showed higher abundance in the rhizospheres of *Arabidopsis halleri* plants that were able to accumulate more Cd (Muehe et al., 2015). The relative abundance of *Nitr ospira* decreased with the increase in Cd (II) concentration, and showed recovery if amendments were applied continuously to Cd-contaminated soil (Wang et al., 2019a; Shi et al., 2019; Wang et al., 2019b). Therefore, the content of *Nitrospira* should be associated with changes in Cd concentration. According to the microbial-community distribution at the genus level in the current study, the abundance of *Nitrospira* in rhizosphere soils was less than that in bulk soil, which was consistent with that the content of Cd in rhizosphere soil was less than that in bulk soil (Fig. S4). Moreover, the proportion of Nitrospirae was decreased in both bulk and rhizosphere soil in response to aggravated Cd pollution (Fig. 4). *Nitrospira* has been known to be aerobic chemolithoautotrophs and oxidise nitrite (Daims, Lücker & Wagner, 2016). It is difficult to determine whether the impact is direct or indirect, and the relationship between *Nitrospira* and Cd should be further studied.

It was reported that Rhizobacteria could induce changes in metal speciation, toxicity, and mobility depending on complex interplay between metal and the (bio)geochemical microclimate in which microorganisms are located (Antoun & Prevost, 2006; Saluja, Gupta & Goel, 2011; Sobariu et al., 2017). Therefore, the future investigation on the distribution of *Nitrospira* and Nitrospirae between rhizosphere soil and bulk soil may provide more information for improving the Cd-resistance of wheat in Cd-polluted field. In addition, a variety of plant-growth regulators, such as inorganic amendments, proper fertilization, organic, silicon, and manure, are usually added to the soil to reduce the uptake of Cd by wheat and rice (Rizwan et al., 2016; Zhang et al., 2018; Wang et al., 2019b). Accordingly, how these plant-growth regulators shape the microbial communities between rhizosphere soil and bulk soil to regulate the solubility of Cd and reduce the wheat uptake should be investigated in future.

### Cadmium-tolerant bacterial groups may provide new resources for wheat-growth regulators to enhance Cd resistance

Bioremediation is an environmentally friendly technology that uses organisms to remove or immobilize heavy metals from a contaminated site. Many strains can be enriched and become major functional populations that are highly tolerant when exposed to heavy metals (Zhang et al., 2012; Nath, Deb & Sharma, 2018). The application of root endophytes and arbuscular mycorrhizal fungi could increase Cd-stressed wheat growth and chlorophyll contents with increased root Cd and decreased shoot Cd, especially under higher Cd concentrations (Shahabivand et al., 2012). Plant-growth-promoting rhizobacteria have been used to decrease Cd uptake and Cd-induced oxidative stress in both wheat and maize (Ahmad et al., 2015; Hassan et al., 2016). Especially, the PGPR with both deaminase and nitrogen fixing activities are more resilient against cadmium pollution than PGPR having either deaminase or nitrogen fixing activity alone in wheat (Hassan et al., 2016). Therefore, the application of microorganisms in bioremediation not only reduce Cd uptake in plants but also promote plant growth.

In the current study, we found that several microbial biomarkers (*Anaeromyxobacter*, *Marmoricola*, *Nocardioides*, and *Haliangium*) showed significant differences in abundance between bulk- and rhizosphere-soil types (Fig. 6). Lin et al. (2020) reported that phytogenic iron oxide nanoparticles (PION) significantly reduced soil Cd availability and Cd in rice shoot, and the abundance of *Anaeromyxobacter* was higher in the rhizosphere soil than in the bulk soil. Ma et al. (2020) found the abundance of *Nocardioides* was significant increased in organic acids treatment of cadmium-contaminated soil and then reduced the Cd toxicity after organic acid used for remediation. As shown in Fig. S7, *Nocardioides* showed significant abundance in rhizosphere in SC soils. Therefore, our results also show that numerous phylogenetic groups at order, family, and genus levels could be significantly distinguished in rhizosphere soil of different contamination levels. Moreover, engineered plant–microbe symbiosis for Cd rhizoremediation could enhance the tolerance of microbes to heavy metals (Wu et al., 2006; Zubair et al., 2016). The discovery of additional microorganisms with greater tolerance in wheat rhizosphere soil will provide more promising strains as resources of new plant regulators for further improving the Cd resistance of wheat and other plants.

### Rhizosphere-microbe-related pollution indices as indicators of probable heavy-metal toxicity

To develop efficient protection and management plans against heavy-metal pollution in addition to providing a theoretical indicator of the levels of hazard and to monitor remediation, the evaluation of heavy-metal pollution-degree indices is needed. Bacteria colonizing the roots of plants have beneficial effects on plant growth and development, helping plants adapt to their environment. In turn, the secretion of plant exudates that contain carbohydrates, amino acids, nutrients, and other growth factors can benefit and stimulate microbial activity (Berendsen, Pieterse & Bakker, 2012; Etesami, 2018). Therefore, soil conditions have a marked impact on the balance between plant and rhizosphere microorganisms. Rhizosphere-microbe-related pollution indices help to determine the probable heavy-metal toxicity for plants. In this study, the richness index (Chao1 value) under severe pollution was less 15% than that of nonpollution conditions. The index of microbial diversity (Shannon value) under severe pollution was 5% lower than that of nonpollution conditions. We propose that the soil was severely polluted when the richness and diversity of microorganisms decreased by 15% and 5%, respectively.

## Conclusions

By comparing and characterizing the bacterial community of rhizosphere and bulk soils in wheat fields under various levels of Cd pollution, we have provided critical field-based evidence that long-term Cd pollution remarkably changes bacterial communities and causes significant loss in the diversity of rhizosphere soil. The observed taxonomic changes may further alter major plant–bacteria interactions. Our findings provide a valuable reference for further attempts to maintain sustainable ecological function or conduct risk evaluation in combination with the determination of heavy-metal Cd levels in crops.

##  Supplemental Information

10.7717/peerj.10302/supp-1 Supplemental Information 1 Map of the town of Xushe in the city of Yixing and location of the sampling station Note: 74 sites were indicated as yellow pentagram. CK (red triangle), VMC (red circle), MC (red diamond), SC (red square).

10.7717/peerj.10302/supp-2 Supplemental Information 2 Rarefaction curves for the Shannon indices in the treatments Error bars indicate means ±  standard error (*n* = 6).

10.7717/peerj.10302/supp-3 Supplemental Information 3 Venn diagrams of shared OTUs (A) Overlapping among bulk soils under various contaminations. (B) Overlapping among rhizosphere soils under various contaminations. OTUs are counted only when they appear at least once in a biological repeat.

10.7717/peerj.10302/supp-4 Supplemental Information 4 P3D PCoA obtained by Bray-Curtis distance matrix showing the separation of bacterial communities with ADONIS statistics *P*-values (*P* < 0.001) among bulk or rhizosphere soils under various pollution levels Four different soil samples were separately colored. (A) Bulk soils. (B) Rhizosphere soils.

10.7717/peerj.10302/supp-5 Supplemental Information 5 Bacterial community composition between the bulk soil and rhizosphere soils based on the relative abundance of top 15 bacterial class under various contamination contents (A) Without cadmium contamination (B) With very mild cadmium contamination (C) With mild cadmium contamination (D) With severe cadmium contamination.

10.7717/peerj.10302/supp-6 Supplemental Information 6 Abundance histogram of major taxonomic units that were significant in rhizosphere soils under various cadmium concentration conditions using linear-discriminant-effect size analysis (LEfSe) (A) Cladogram of bacterial communities among rhizosphere soils under various cadmium concentration conditions. Small circles and colored areas represent the abundance of taxa in each respective group (red, bulk soil; green, rhizosphere soil). Yellow circles, indicate non-significant differences in abundance between both soil samples for a particular taxonomic group. (B) LDA scores of biomarker bacteria, shown as horizontal bars for biomarker bacteria with an LDA score (*log*10) > 4.

10.7717/peerj.10302/supp-7 Supplemental Information 7 Abundance histogram of major taxonomic units that were significant in bulk soils under various cadmium concentration conditions using linear-discriminant-effect size analysis (LEfSe) (A) Cladogram of bacterial communities among bulk soils under various cadmium concentration conditions. Small circles and colored areas represent the abundance of taxa in each respective group (red, bulk soil; green, rhizosphere soil). Yellow circles, indicate non-significant differences in abundance between both soil samples for a particular taxonomic group. (B) LDA scores of biomarker bacteria, shown as horizontal bars for biomarker bacteria with an LDA score (*log*10) > 4.

10.7717/peerj.10302/supp-8 Supplemental Information 8 Locations and soil samples characteristics in bulk soils

10.7717/peerj.10302/supp-9 Supplemental Information 9 Cd concentration in all soil samples

10.7717/peerj.10302/supp-10 Supplemental Information 10 Sequencing statistics

## Additional Information and Declarations

### Field Study Permissions

The following information was supplied relating to field study approvals (i.e., approving body and any reference numbers): Prior to the start of the project, permission was obtained from the landowner (Zuliang Jiang, Jiang Farms, Yifeng Village, YiXing) to access the site and monitor soil quality for the duration of the project.

### Data Availability

The following information was supplied regarding data availability: The data corresponding to raw reads of our 16S rRNA gene sequencing are available at SRA: PRJNA661129.
