# Supplementary figures and images for: Characterization and comparison of the bacterial communities of rhizosphere and bulk soils from cadmium-polluted wheat fields
Source: PeerJ. 2020 Nov 4;8:e10302. doi: 10.7717/peerj.10302 (PMC7648459; doi:10.7717/peerj.10302)

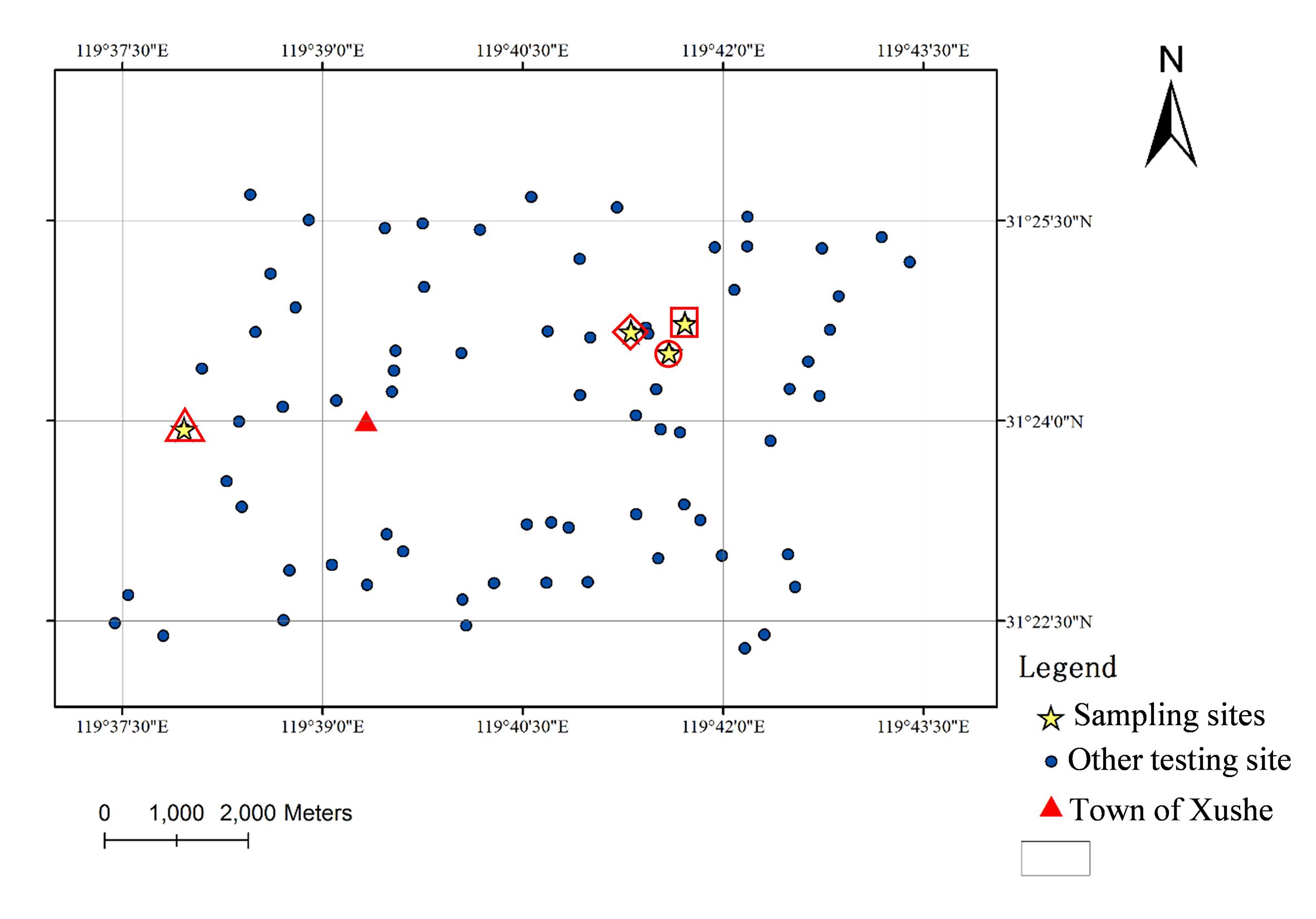

Supplement: Supplemental Information 1 — Note: 74 sites were indicated as yellow pentagram. CK (red triangle), VMC (red circle), MC (red diamond), SC (red square). [file peerj-08-10302-s001.png]

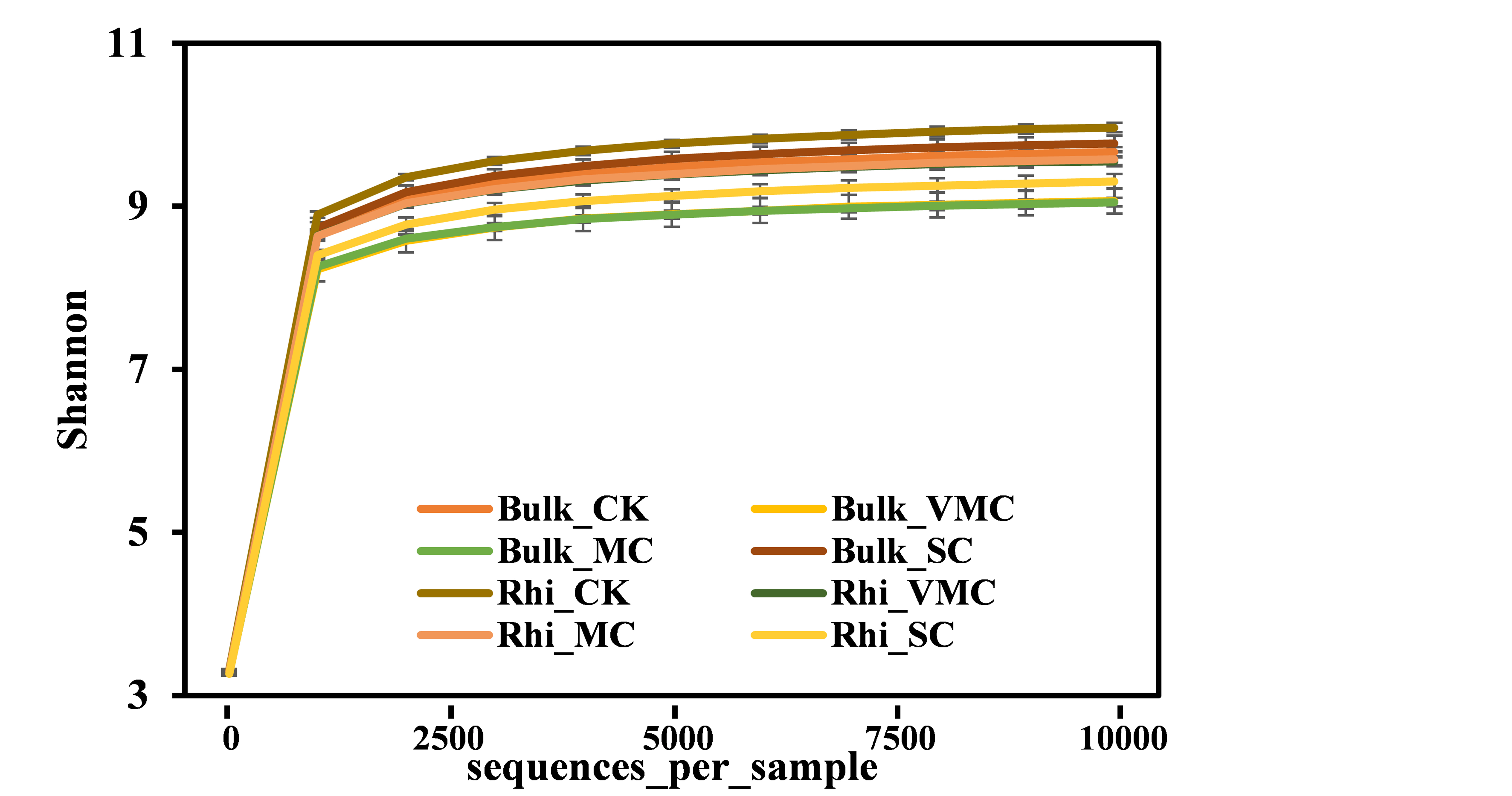

Supplement: Supplemental Information 2 — Error bars indicate means ± standard error (n = 6). [file peerj-08-10302-s002.jpg]

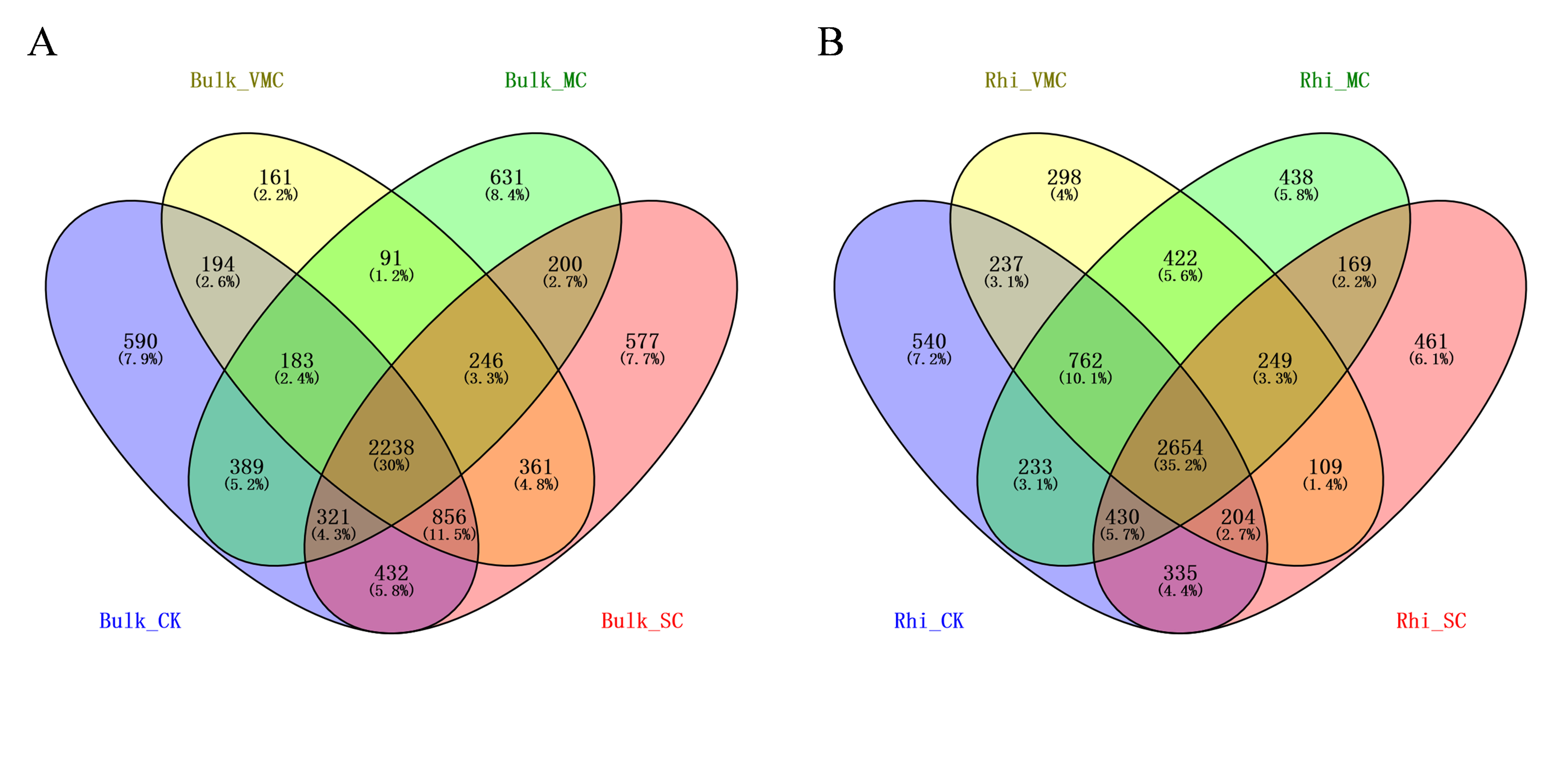

Supplement: Supplemental Information 3 — (A) Overlapping among bulk soils under various contaminations. (B) Overlapping among rhizosphere soils under various contaminations. OTUs are counted only when they appear at least once in a biological repeat. [file peerj-08-10302-s003.jpg]
