# Supplementary material for: Characterization and comparison of the bacterial communities of rhizosphere and bulk soils from cadmium-polluted wheat fields
Source: PeerJ. 2020 Nov 4;8:e10302. doi: 10.7717/peerj.10302 (PMC7648459; doi:10.7717/peerj.10302)
Supplement: Supplemental Information 8 [file peerj-08-10302-s008.docx]

**Table S1. Locations and soil samples characteristics in bulk soils.**

| **Sample Name** | **Location**  **(longitude)** | **Location**  **(latitude)** | **CEC^a^**  **(cmol kg ^−1^ )** | **TP^b^  (g kg ^−1^ )** | **AP^c^  (g kg ^−1^ )** | **TN^d^  (g kg ^−1^ )** | **HN^e^  (g kg ^−1^ )** | **pH** | **SOM^f^  (g kg ^−1^ )** |
| --- | --- | --- | --- | --- | --- | --- | --- | --- | --- |
| Bulk_CK | 119.63277 | 31.39900 | 15.87±3.01 | 0.087±0.011 | 32.8±1.6 | 0.200±0.035 | 349±65 | 6.55±0.82 | 42.6±3.4 |
| Bulk_VMC | 119.69524 | 31.41221 | 20.10±2.83 | 0.088±0.013 | 32.0±4.0 | 0.202±0.017 | 209±59 | 5.57±0.21 | 37.9±2.9 |
| Bulk_MC | 119.68853 | 31.41118 | 19.08±0.19 | 0.126±0.044 | 50.3±3.9 | 0.159±0.018 | 122±34 | 5.44±0.11 | 24.8±2.1 |
| Bulk_SC | 119.69327 | 31.40853 | 20.86±4.86 | 0.047±0.004 | 9.83±1.3 | 0.105±0.017 | 155±26 | 6.79±0.31 | 18.5±1.7 |

**^a^** Cation exchange capacity

**^b^** Total phosphorus

**^c^** Available phosphorus

**^d^** Total nitrogen

**^e^** Hydrolyzable nitrogen

**^f^** Soil organic matter

Values are means ± standard deviation (n = 6).
