# Supplementary material for: Characterization and comparison of the bacterial communities of rhizosphere and bulk soils from cadmium-polluted wheat fields
Source: PeerJ. 2020 Nov 4;8:e10302. doi: 10.7717/peerj.10302 (PMC7648459; doi:10.7717/peerj.10302)
Supplement: Supplemental Information 9 [file peerj-08-10302-s009.docx]

**Table S2. Cd concentration in all soil samples**

| **Sample Name** | **exchangeable -Cd**  **(mg kg ^−1^)** | **Total Cd**  **(mg kg ^−1^)** | **exchangeable -Cd**  **vs Total Cd** |
| --- | --- | --- | --- |
| Bulk_CK | 0.15±0.03 | 0.25±0.05 |  |
| Bulk_VMC | 0.30±0.04* | 0.60±0.14* |  |
| Bulk_MC | 0.68±0.01* | 1.31±0.27* | * |
| Bulk_SC | 7.28±1.26 ** | 14.64±2.01** | ** |
| Rhizosphere_CK | 0.07±0.01 | 0.17±0.01 |  |
| Rhizosphere_VMC | 0.27±0.05* | 0.63±0.17* |  |
| Rhizosphere_MC | 0.61±0.11* | 1.42±0.30* | ** |
| Rhizosphere_SC | 3.85±0.52** | 9.72±1.91** | ** |

**p*<*0*.05, ***p*<*0*.01.
