# Supplementary material for: Characterization and comparison of the bacterial communities of rhizosphere and bulk soils from cadmium-polluted wheat fields
Source: PeerJ. 2020 Nov 4;8:e10302. doi: 10.7717/peerj.10302 (PMC7648459; doi:10.7717/peerj.10302)
Supplement: Supplemental Information 10 [file peerj-08-10302-s010.docx]

**Table S3. Sequencing statistics**

| **Sample** | **Clean_tags** | **Valid_tags** | **Valid_percent** | **PD_whole_tree** | **Goods_coverage** | **Observed_species** |
| --- | --- | --- | --- | --- | --- | --- |
| Bulk_CK | 37595 | 29712 | 79.0% | 75.35 | 0.91 | 2004 |
| Bulk_VMC | 30645 | 24465 | 79.8% | 66.01 | 0.93 | 1695 |
| Bulk_MC | 36062 | 28703 | 79.6% | 64.21 | 0.93 | 1625 |
| Bulk_SC | 32985 | 25989 | 78.7% | 79.75 | 0.91 | 2130 |
| Rhizosphere_CK | 32522 | 25402 | 78.0% | 77.90 | 0.91 | 2153 |
| Rhizosphere_VMC | 42031 | 33437 | 79.4% | 69.50 | 0.92 | 1863 |
| Rhizosphere_MC | 47811 | 38260 | 80.0% | 72.34 | 0.91 | 1924 |
| Rhizosphere_SC | 38945 | 30150 | 77.3% | 66.52 | 0.92 | 1835 |
